# The Ophthalmoscope

**Published:** 1874-04

**Authors:** 


### THE OPHTHALMOSCOPE.

Did you ever look under your house, or peer into a dark cellar, while holding a lamp in your hand, and see a pair of green or red balls of light glaring at you? Perhaps you have, and have recognized them as belonging to your cat or dog, whose eyes you had illuminated in this manner, without knowing that you were performing an ophthalmoscopic examination, although in a very imperfect manner.

The ophthalmoscope is a wonderful instrument, and has done more toward revealing many mysteries and diseases connected with the eye, than all that had previously been discovered in this direction. Its introduction has completlely revolutionized the practice of ophthalmic medicine and surgery, and has made possible the cure of many defects of vision which were entirely beyond our reach, from want of a proper understanding of the internal appearance of the eye in health and disease.

It is now pretty generally known by the public that the opthalmic surgeon has the entire internal arrangement of the eye at his command, that, by the use of the ophthalmoscope, the eye can be so illuminated and scrutinized, as to enable him to detect and recognize diseases at any point of its internal structure. The optic nerve, where it enters the eye, can be observed, its appearance revealing to the skillful oculist, not only conditions that affect the vision, but disturbances of the brain, as well. Insanity, softening of the brain, brights' disease of the kidneys, and many other maladies are at once recognized from the characteristic appearances of the optic disk, when examined by the ophthalmoscope. The *retina*, that sensitive, nervous expansion, at the very back portion of the eye-ball, beyond and behind the pupil, completely out of reach of any scrutiny from the unaided eye, and which is the seat of so very many diseases and disturbances, that result in total blindness, and which were always classed under the head of "amurosis," a condition which has been aptly explained as "a disease in which the patient sees nothing, and the doctor, too;" all this, is at once revealed and explained, under the magic influences of the ophthalmoscope.

This instrument, to which the oculist is so much indebted for the valuable information that has been given us by scientific oculists, during the past ten years, was invented by Prof. Helmholz of Vienna, in 1851, and first given to the scientific world in the publication of a little pamphlet entitled, "Description of a Mirror to Examine the Retina of the Living Eye."

Prof. Helmholtz had long recognized the great want of such an instrument, and was induced to experiment in this direction by observing that the pupil of a cat's eye appeared red when illuminated, in a dark room, by a bright light. Knowing that the pupil *should* appear black—as it is simply a hole in a curtain that excludes the light from entering in any other direction—and, knowing that the interior of the eye is lined with a black pigment, which renders the chamber dark, and therefore, the pupil black; he wisely concluded that the red appearance of the pupil of the cat's eye under illumination, must have been due to the light reflected from the blood vessels distributed upon the back portion of the animal's eye, to his own. Experiments with various devices, bits of glass, etc., for reflecting a strong light through the pupil, confirmed his belief; and finally led, after much study and experiment, to overcome many difficulties of refraction and reflection of light, to the present ophthalmoscope, now universally used by all ophthalmic surgeons.

It is simply a small concave mirror, of highly polished steel; in the centre is a small aperture, through which the surgeon looks into the eye to be examined. The patient is seated by a table, in a perfectly dark room.—A lamp is placed immediateiy back of, and at one side, his head. This light is so constructed and shaded as not to illuminate the room nor the patient's face. The oculist sits facing the patient, receives the rays of light from the lamp, upon the concave mirror, and reflects it into the patient's eye, through the pupil, and in the same manner as you have seen little boys reflect sunlight into each other's faces from a fragment of broken mirror. A convex lens is held with one hand near the patient's eye, in order to concentrate the illuminating rays upon the *retina*, while, at the same time, it magnifies the points under examination. In this way, every portion of the internal eye is examined, *and can be seen, too*, as plainly and distinctly as the lines upon the palm of your hand. Enlargement or congestion of the retinal vessels; rupture of these vessels, with escape of blood under the retina or into the humors of the eye, giving rise to dark spots or blotches, or flying specks before the eyes; separation of the retina from its proper attachments, atrophy or shrinking of the retina, causing blindness in some portion of the eye, with one-half or one-quarter of the eye seeing properly and the balance blind; tumors growing from the internal structures of the eye; inflammation of the parts; opacity or haziness of the lens, or vitrious humor; all these, and many more difficulties, are at once revealed by the ophthalmoscope. Anomlies of refraction are also detected by this instrument; and glasses can be fitted to eyes requiring them, accurately, by its means.

In short, the wonders that have been and are constantly being accomplished through the instrumentality of this ingenious little device, surpasses anything ever before presented to the scientific surgeon. Since its introduction it has been so modified as to afford means for examining the throat, speaking apparatus, nose and ear; while instruments are constantly appearing for the examination of almost every organ of the body, all based upon the principle of the ophthalmoscope.

The application of the ophthalmoscope to the eye is not painful, as the most sensitive eyes are readily examined without difficulty.

We hope we have written sufficient to induce any of our readers suffering from any difficulty of the eye, to lose no time in having the cause revealed under the searching influences of the ophthalmoscope.

